# Diagnostic and therapeutic potential of circular RNA in brain tumors

**DOI:** 10.1093/noajnl/vdad063

**Published:** 2023-06-13

**Authors:** Keisuke Katsushima, Kandarp Joshi, Ranjan J Perera

**Affiliations:** Department of Neurosurgery and Oncology, Johns Hopkins University School of Medicine, Baltimore, Maryland, USA; Cancer and Blood Disorders Institute, Johns Hopkins All Children’s Hospital, Florida, USA; Department of Neurosurgery and Oncology, Johns Hopkins University School of Medicine, Baltimore, Maryland, USA; Cancer and Blood Disorders Institute, Johns Hopkins All Children’s Hospital, Florida, USA; Department of Neurosurgery and Oncology, Johns Hopkins University School of Medicine, Baltimore, Maryland, USA; Cancer and Blood Disorders Institute, Johns Hopkins All Children’s Hospital, Florida, USA

**Keywords:** circRNA, glioma, medulloblastoma, microRNA

## Abstract

Circular RNAs (circRNAs) are a class of RNA with a stable cyclic structure. They are expressed in various tissues and cells with conserved, specific characteristics. CircRNAs have been found to play critical roles in a wide range of cellular processes by regulating gene expression at the epigenetic, transcriptional, and posttranscriptional levels. There is an accumulation of evidence on newly discovered circRNAs, their molecular interactions, and their roles in the development and progression of human brain tumors, including cell proliferation, cell apoptosis, invasion, and chemoresistance. Here we summarize the current state of knowledge of the circRNAs that have been implicated in brain tumor pathogenesis, particularly in gliomas and medulloblastomas. In providing a comprehensive overview of circRNA studies, we highlight how different circRNAs have oncogenic or tumor-suppressive roles in brain tumors, making them attractive therapeutic targets and biomarkers for personalized therapy and precision diagnostics. This review article discusses circRNAs’ functional roles and the prospect of using them as diagnostic biomarkers and therapeutic targets in patients with brain tumors.

**Key Points**

1. CircRNAs are aberrantly expressed in brain tumors.
2. CircRNAs are involved in brain tumor progression, invasion, and migration.
3. CircRNAs have potential to be useful as diagnostic biomarkers and therapeutic targets.

The human genome can be divided into 2 broad categories of sequences: (1) A minor group of protein-coding genes, accounting for perhaps 20,000 genes or 2% of the entire genome; and (2) a major group of sequences that encode noncoding RNA (ncRNA) molecules, which are not translated into proteins. ncRNAs regulate the translation of other RNAs and control the production of functional proteins from protein-coding RNAs. Our understanding of the human transcriptome increased significantly by learning the role of regulatory ncRNAs in many diseases, including brain tumors.^1–7^ Among the ncRNAs, circular RNAs (circRNAs) have attracted intense research scrutiny in recent years. These circRNAs are single-stranded, covalently closed continuous loop structures lacking free ends and a polyadenylate tail. Approximately 15% of active genes in the human genome can potentially give rise to circRNAs.^8^ Exonic and intronic sequences constitute circRNAs. They are primarily generated by backsplicing, a noncanonical alternative RNA splicing event mediated by the spliceosome and regulated by a combination of cis-elements and trans-factors.^8,9^ Due to the absence of free ends, circRNAs are not susceptible to destruction by RNA degradation machinery and are more stable than linear RNAs.^10^ Some circRNAs regulate transcription and splicing and may also be translated to polypeptides. circRNAs regulate cancer hallmarks such as growth signals, proliferation, angiogenesis, anti-apoptosis, unlimited replicative potential, and metastasis.

Gliomas are the most common primary type of adult brain cancer, consisting of up to 80% of malignant brain tumors.^11^ There have been substantial efforts to characterize the gene expression, mutational, and epigenetic landscapes of gliomas.^12^ Low-grade glioma (LGG) contains CNS WHO grades 1 and 2, while high-grade glioma (HGG) contains CNS WHO grades 3 and 4. LGG, accounting for 6% of CNS primary tumors in adult, usually presents a more promising prognosis.^11^ It is also important that the mutational status of isocitrate dehydrogenases 1 and 2 (IDH1 and IDH2) should be considered regarding LGG and HGG.^13^ Except for IDH status, O-6-methylguanine-DNA-methyltransferase (MGMT) methylation is hitherto regarded as another significantly prognostic biomarker. Methylation of the MGMT promoter has been observed in approximately 50% of grade 4 gliomas, commonly referred to as glioblastoma multiforme (GBM).^14^ MGMT status holds strong prognostic value and potential predictive information on the benefit of alkylating chemotherapy.^15^

In children, medulloblastomas (MBs) are the most common brain tumors, representing approximately 20% of pediatric brain tumors but only 1% of adult cases.^11^ Transcriptional programs in MBs mimic developmental cerebellar lineages, highlighting their embryonic origin.^16^ The clinical management of MBs depends on several factors, including molecular and histopathological tumor subtype, stage and extent of resection, location, and overall patient health. Treatment strategies tend to be aggressive, consisting of surgical resection, radiotherapy, chemotherapy, and stem cell/bone marrow transplantation. Despite advances in their diagnosis and treatment, MB remains deadly in 35%–40% of cases. MBs are subdivided into the following major molecular subgroups^17^: Wingless-type (WNT)-activated MBs and sonic hedgehog (SHH)-activated MBs; and 2 provisional non-WNT/SHH subgroups: Group 3 MBs (25%; poor prognosis, infants and children) and group 4 MBs (35%; intermediate prognosis, children and adults).^18,19^

Studies have shown that circRNAs are involved in brain tumor development due to their highly stable structure and abundance in tumor tissues.^20–24^ Their altered expression is expected to become a new marker for early diagnosis and prognostic assessment of brain tumors or a new target for effective treatment. In this review, we explore the expression profiles and functions of circRNAs in brain tumors, especially gliomas and MBs, the most common malignant tumors of the central nervous system,^25^ and discuss their potential use as diagnostic and prognostic biomarkers and therapeutic targets.

## Biological Functions of Circular RNAs in Brain Tumors

**Figure 1.**
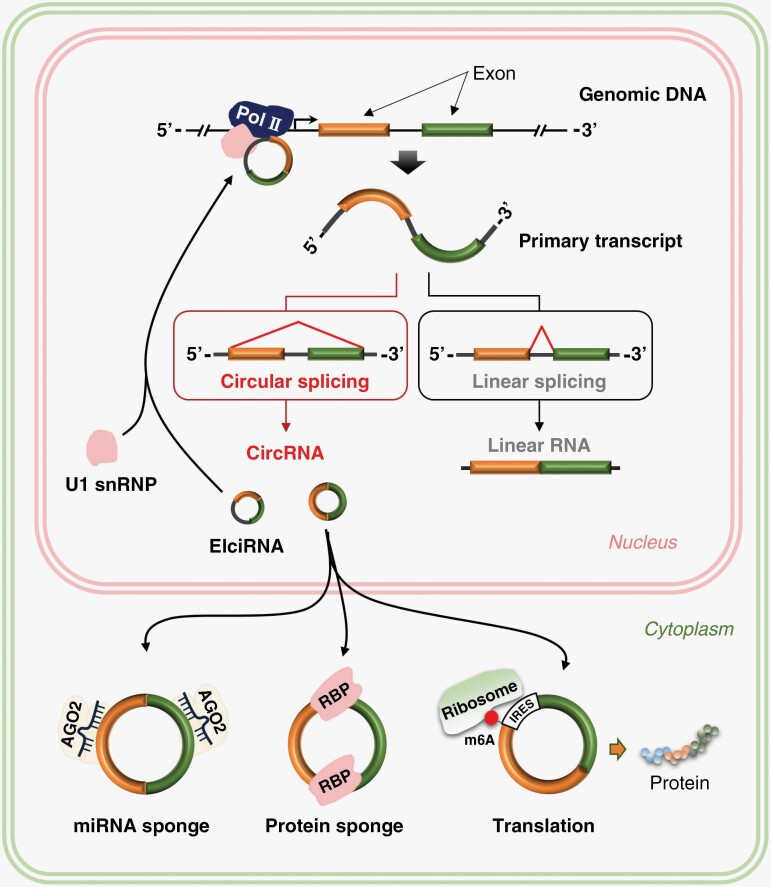
Biogenesis and function of circular RNAs (circRNAs). Linear splicing removes introns from primary transcript to generate mature linear mRNA. Circular splicing removes introns from the primary transcript to generate a stable circRNA. Exons and introns of the primary transcript are circularized into elciRNAs. CircRNAs can act as miRNA sponges and subsequently regulate relevant target gene expression. CircRNA can regulate transcription and splicing of their parental genes. CircRNA can bind to several proteins to mediate their actions. Abundant internal ribosome entry site (IRES) and N6-methyladenosine modification (m6A) modification can promote multiple circRNAs to translation.

Because circRNAs are rich in miRNA binding sites, circRNAs are functionally diverse and often function as microRNA (miRNA) sponges. In addition, circRNAs have roles in regulating parent gene expression, regulating parent gene selective splicing, protein translation, and participating in intercellular communication by entering exosomes (Figure 1).

### CircRNA–miRNA Biomarkers in Brain Tumors

Through miRNAs response elements, ncRNA s and coding RNAs form a large-scale regulatory network in the transcriptome. Interestingly, circRNAs contain particular miRNA response elements (MREs), which provide the structural basis for their function as molecular sponges. CircRNAs can function as competing endogenous RNA (ceRNAs) or miRNA sponges to inhibit miRNA and up-regulate the expression of target genes by MREs. Hsa_circ_0043280 can downregulate PAQR3 by competing with miR-203a-3p and inhibiting tumor growth.^26^ Hsa_circ_0006349 promotes MKP1 expression by blocking miR-98, which enhances cell proliferation and glycolysis and promotes the malignant progression of tumors.^27^

### Interaction with CircRNA and RNA-Binding Proteins in Brain Tumors

RNA-binding proteins (RBPs) can bind to double- or single-stranded RNA. RBPs participate in biological processes, including RNA splicing, processing, localization, and transport. Muscleblind (MBL) is a muscleblind-like 1 (mbnll). MBL promotes the production of circ-Mbl, which has a specific MBL binding site, and circ-Mbl has a robust direct interaction with MBL protein.^9^ circ-Mbl regulates MBL protein and reduces its own mRNA production by promoting circ-Mbl production when MBL is in excess. circ-Mmbl can also eliminate excess MBL by binding to MBL.^28^ CircRNA can facilitate the interaction between DNA, RNA, and RBP to perform biological functions by binding to related proteins.^29^ Liu et al. found that splicing factor SRSF10 can bind to Alu elements on both sides of the circ-ATXN1 pre-mRNA,^30^ thus promoting the generation of circ-ATXN1 and the proliferation, migration, and tube-forming capacity of glioma-exposed endothelial cells (GECs) via circ-ATXN1/miR-526b-3p/MMP2/VEGFA pathway. He et al. demonstrated that FUS binds to circ_002136, which positively regulates the SOX13 transcription factor by sponging miR-138-5p. SOX13 promotes SPON2 expression by combining the SPON2 promoter region and activates the FUS promoter to form a feedback loop that promotes the viability and antigenic capacity of GECs.^31^ In contrast, knockout of FUS and circ_002136 can reduce the tube-forming ability of GECs. In addition, a recent study demonstrated that the abundance of RBPs increases with the glioma grade.^32^ There may be several roles for circRNA-RBP interactions. CircRNAs can control gene expression by binding to cis-elements to regulate transcription factors or modulate the epigenetics gene regulation. Since circRNAs also form an RBP-circRNA-mRNA ternary complex, such interactions provide a clear molecular mechanism of circular RNA function in brain tumors.

### Involved in Intercellular Communication via Extracellular Vesicles

The primary function of extracellular vesicles (EVs) is intercellular communication through their contents under physiological and pathological conditions.^33^ Tumor cells can secrete more EV than normal cells with some variation in contents, and tumor cell-derived EV can provide a suitable microenvironment for tumor development, such as cell proliferation, angiogenesis and metastasis, and drug resistance.^34^ Also, circRNAs can be included in EV and thus participate in tumorigenesis and progression. Zhao et al. found that circRNA-ATP8B4 from radioresistant GBM-derived EVs sponged miR-766 to facilitate glioma cell radioresistance.^35^ Further, miR-21 levels in cerebrospinal fluid (CSF)-derived EVs from GBM patients were significantly higher than in controls, while there was no difference in serum-derived EVs miR-21 expression.^36^ These studies suggested that CSF-derived EV circRNAs were promising biomarkers for glioma diagnosis and prognosis. EVs’ phospholipid bilayer membrane structure protects circRNA because of its resistance to ribonuclease degradation; circRNAs could be selectively packaged into EVs. Additionally, the biocompatibility of EVs can overcome the blood-brain barrier. Lai et al. developed an EVs reporter system that can monitor the EVs biodistribution over time in vivo imaging. They found that systemic injection of EVs reached the tumor sites within an hour.^37,38^ Therefore, EVs circRNAs hold great potential for clinical diagnosis and treatment of Glioma.

### Peptide Translation

Normally, circRNAs cannot be flipped, but studies have found that exon sequences of some circRNAs can be translated into proteins.^39^ Some circRNAs contain internal ribosome entry site (IRES) sequences, which can bind directly to ribosomes, and can be translated in eukaryotic cells.^40^ The 40S subunit of eukaryotic ribosomes binds to circRNAs and can directly initiate translation.^41^ Moreover, circRNAs may also encode proteins through an alternative mechanism driven by the N6-methyladenosine modification (m6A).^42^ This kind of modification preferentially appears in the long exon regions of circRNAs and is enriched around the upstream and middle exon regions.^43^ m6A could methylate circRNAs with the help of the METL3/METTL14 complex.^42^ And circRNAs with m6A-induced ribosome engagement sites (MIRES) could initiate the translation process by recruiting YTHDF3, which can recruit other translation initiation factors, including eIF4G2.^44^ An ORF in circ-SHPRH can encode a functional protein (SHPRH-146aa) in an IRES-driven way.^45^ SHPRH-146aa overexpression can also induce PCNA degradation, which can be inhibited by the proteasome inhibitor MG132. SHPRH-146aa may increase SHPRH level by extending its half-life and protecting full-length SHPRH from denticle-less E3 ubiquitin protein ligase-mediated degradation, thereby promoting PCNA degradation and inhibiting cell growth and tumorigenesis.

### Regulation of Gene Expression

CircRNAs can interact with RNA to participate in posttranscriptional regulation. CircRNAs are formed with a balance between competitive complementary pairing between introns and linear RNAs, which affects mRNA expression and translation.^46^ The ORF-containing circRNAs produced by COL6A3 encode a novel 198-aa functional peptide, and hsa_circ_0006401–198-aa promotes the stability of the host gene COL6A3 mRNA, thereby facilitating cell proliferation and translocation.^47^ CircRNAs can regulate the transcription of disease-related parent genes, affecting the expression of the parent gene and its target genes. This transcription regulation offers new ideas for treating gene-related diseases. Some intron-derived circRNAs are mainly localized in the nucleus and can interact with RNA polymerase II to promote transcription of their own coding genes.^48^ EIciRNA (circRNA with circularized exons and introns of primary transcript) predominantly localizes in the nucleus and affects parental gene expression^48^(Figure 1). ci-ankrd52 is abundant at the transcriptional loci of its host gene (ANKRD52) and plays a cis-regulatory role on the transcriptional levels by positively modulating the pol II complex.^49^ The formation of EIciRNA–U1 snRNP complexes mediates the transcription-enhancing effects of EIciRNAs. These complexes may interact with the Pol II transcription complex.^48^

## Mechanistic and Functional Role of CircRNAs in Brain Tumors

**Figure 2.**
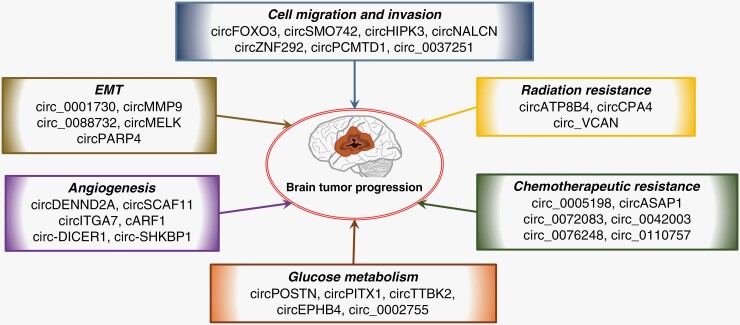
Functional role of circular RNAs (circRNAs) in brain tumors. Selection of circRNAs involved in brain tumor cell migration, invasion, EMT, `angiogenesis, glucose metabolism, angiogenesis, radiation resistance, and chemotherapeutic resistance. EMT, epithelial-mesenchymal transition.

Overall, circRNAs can modulate the classic miRNA–mRNA axis and participate in tumor-related signaling pathways, enhancing cell growth, invasion, and metastasis. Exploring the cancer-promoting mechanisms of circRNA is valuable for understanding the molecular biology of brain tumors and developing new targeted therapies. Here we discuss recently reported mechanistic insights into how circRNAs regulate gene expression and contribute to tumor formation. Figure 2 summarize the circRNAs implicated in brain tumors and their roles in cell proliferation, growth suppression, migration, invasion, and metastasis.

### CircRNAs Regulate Brain Tumor Progression

circEPHB4 is upregulated in Glioma and increases SOX10 and Nestin expression levels by competitively binding to miR-637, which ultimately stimulates tumor cell stemness and self-renewal.^50^ The self-renewal capacities of glioma stem cells (GSCs) contribute to tumor proliferation and recurrence. Recent studies found that circATP5B and circCHAF1A were upregulated in GSCs, and promoted GSC proliferation through miR-185-5p/HOXB5 and miR-211-5p/HOXC8 axes, respectively.^51,52^ In addition, circRNA can mediate the cell proliferation of Glioma through activation of EGFR–STAT3 signaling. Circ-E-Cad is selectively expressed in GBM and encodes E-cadherin protein variant (C-E-Cad). Circ-E-Cad is a secretory protein that activates the oncogenic EGFR signaling pathway by directly binding and activating EGFR signaling. Interestingly, circ-E-Cad RNA is an oncogenic circRNA and an independent prognostic factor in GBM.^23^

### CircRNAs Regulate Brain Tumor Cell Migration and Invasion

circFOXO3 can serve as a competitive ceRNA to upregulate NFAT5 by sponging miR-138-5p and miR-432-5p, which enhance glioma cell migration and invasion.^53^ Additionally, circSMO742 contributes to mediating cell proliferation and invasion by targeting miR-338-3p and upregulating smoothened (SMO) expression levels.^54^ A recent study showed that circNALCN was downregulated in Glioma and inhibited tumorigenesis and invasion by targeting miR-493.^55^ circZNF292 can interact with other related genes, including cyclin A, VEGFR, and EGFR, to promote tumorigenesis and invasion. Silencing circZNF292 can block the cell cycle in the S/G2/M phase, inhibiting glioma cell migration and tube formation.^56^ Further, circPCMTD1 and hsa_circ_0037251 promote glioma cell proliferation and metastasis by regulating the mammalian target of rapamycin (mTOR) pathway via sponging of miR-224-5p and miR-1229-3p, respectively.^57^ Oncogenic circRNAs have also been reported in MBs, circ-SKA3, circ-DTL, and circ_63706 promoted the proliferation, migration, and invasion of medulloblastoma cells by regulating gene expression.^58,59^

### CircRNAs Regulate Epithelial–Mesenchymal Transition

Epithelial–mesenchymal transition (EMT) is a process by which cells lose their polarity and acquire the ability to migrate, invade, and metastasize. circ_0001730 positively modulates the Wnt/β-catenin pathway, which can induce tumor cell invasion and migration and the EMT process in glioblastoma cells.^60^ A study has reported that eIF4A3-induced circMMP9 acts as miR-124 sponges to upregulate CDK4 and AURKA, promoting glioma cell proliferation and invasion. Interestingly, the circMMP9/miR-124 axis regulates the expression of EMT markers in glioma cells.^61^ In addition, hsa_circ_0088732, derived from the cyclization of Lcn2, accelerates glioma progression, migration, invasion, and EMT through the miR-661/RAB3D axis.^62^ circ-PTN is an oncogenic factor. Its overexpression promotes Sox9 and ITGA5 by downregulating miR-145-5p and miR-330-5p, increasing glioma proliferation, stemness, and self-renewal.^63^ circMELK and circPARP4 regulate GBM EMT and stemness of GSCs by upregulating oncogenic proteins EphB2 and FUT4, acting as sponges for miR-593 and miR-125a-5p, respectively.^64,65^

### Angiogenesis is Driven by CircRNAs in Brain Tumors

Cancer and stromal cells usually lead to nutrient and oxygen limitation, thus establishing anoxic microenvironments. Hyperosmotic inducible factor 1α (HIF1α) is a hypoxia marker that greatly influences malignant transformation and tumor metastasis.^66^ circDENND2A, derived from the DENND2A gene, is highly expressed in HIF1α-associated glioma cells and facilitates tumor cell aggressiveness by competitive binding to miR-625-5p.^67^ Further, Jiang et al. recently discovered that circRNA ARF1 (cARF1) upregulates ISL2 by sponging miR-342-3p in GSCs. ISL2 facilitates the angiogenesis, proliferation, and invasiveness of human brain microvessel endothelial cells (hBMECs) via VEGFA-mediated ERK signaling. Interestingly, U2AF2, which ISL2 upregulates, can bind to cARF1 and promotes its stability and expression, forming a feedback loop in GSCs. Overexpressed circ-DICER1 in GECs can act as sponges for miR-103a-3p and miR-382-5p, inducing the upregulation of the downstream target Hsp90β by weakening the inhibitory effect on the ZIC family member 4 (ZIC4) and activates the PI3K/AKT pathway to mediate GEC angiogenesis.^68^

### CircRNAs Regulate Glucose Metabolism

Aerobic glycolysis, also known as the “Warburg effect,” refers to the catabolism process in which tumor cells consume glucose and produce a large amount of lactic acid even when the oxygen supply is sufficient.^69^ Compared to normal brain, Glioma is characterized by increased aerobic glycolysis, leading to hypoxic local tissue, production of HIF-1α and TGF-β, activation of immunosuppressive CD4+ T cells, and inhibition of NK cell activity. These conditions create an acidic, hypoxic, and immunosuppressive TME conducive to malignant invasion, metastasis, and immune resistance. Emerging studies revealed that miRNA might modulate glycolysis by manipulating the expression and activity of glycolytic transporters and rate-controlling enzymes, including hexokinase (HK), 6-phosphate fructokinase, and pyruvate kinase.^70^ Because circRNA may act as a sponge for miRNA, researchers speculate that circRNA may indirectly participate in tumor metabolism. In addition, researchers cannot exclude the possibility that circRNA may directly target these enzymes. circPOSTN was overexpressed in glioma tissues and induced tumor cell proliferation by targeting the miR-361-5p/TPX2 axis.^71^ A study showed that circPOSTN or TPX2 knockdown could inhibit HK2 expression levels, indicating that circPOSTN might be involved in glioma progression by affecting aerobic glycolysis.^71^ Therefore, inhibition of aerobic glycolysis may be a promising anti-tumor therapy. However, studies on metabolism-related circRNAs in Glioma are still limited, and further investigation is warranted.

### CircRNAs Regulate Chemotherapeutic and Radiation Resistance

Temozolomide (TMZ) is a first-line chemotherapeutic drug for high-grade glioma patients, especially GBM, following surgery to prevent glioma recurrence and prolong patient survival. circ_0072083 and hsa_circ_0042003 could be detected in exosomes from tumor tissue. Exosomal circ_0072083/hsa_circ_0042003 can increase TMZ resistance and act as promising therapeutic targets in Glioma.^72,73^ Downregulation of hsa_circ_0076248 or upregulation of its binding miR-181a not only suppressed cell proliferation and migration but also significantly increased the sensitivity of glioma cells to TMZ.^74^ Additionally, hsa_circ_0110757 showed high expression in TMZ-resistant glioma cells, and hsa_circ_0110757 knockdown enhanced the chemosensitivity of glioma cells to TMZ via targeting miR-1298-5p/ITGA1.^75^ circATP8B4 and circCPA4 are overexpressed in Glioma and reduce radiation sensitivity of glioma cells by serving as sponges for miR-766 and miR-760, respectively.^35,76^ Studies have shown that circ_VCAN is expressed at a higher level in radioresistant glioma cells compared to sensitive cells. Further, circ_VCAN overexpression accelerates carcinogenesis and reduces the radiosensitivity of gliomas by regulating miR-1183.^77^

## The Potential Application of CircRNAs in Anti-Tumor Therapy

**Figure 3.**
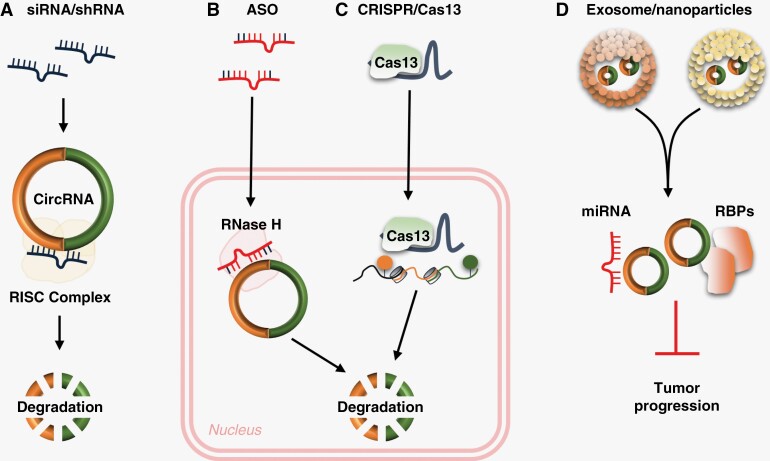
The potential application of circular RNAs (circRNAs) in anticancer therapy. **A** Exogenous siRNA/shRNA activates RNA-induced silencing complex (RISC) and interacts with its specific target circRNA to induce degradation. **B** Exogenous antisense oligodeoxynucleotide (ASO) activates RNase H to induce circRNA degradation. **C** Knockout of circRNA-associated genomic DNA using clustered regularly interspaced short palindromic repeats (CRISPR)/Cas13. **D** The loss of specific circRNAs can be rescued by reintroducing exosomal or nanoparticles’ circRNA into the cells as miRNA or RNA-binding protein (RBP) sponges to inhibit tumor progression.

As reviewed in the previous sections, circRNAs play vital roles in several physiological and pathophysiological processes linked to brain tumors. Therapeutic strategies targeting circRNAs could provide a new perspective to cure brain tumors. Various oligonucleotides have been approved for clinical trials, the most successful of which is a cancer therapy based on interfering RNA (siRNA) or antisense oligonucleotide (ASO). Trabedersen (OT-101), based on ASO, has been approved for the treatment of brain tumors treatment and is currently undergoing Phase III clinical trials. Although no preclinical report exists on using circRNAs alone as a therapeutic target, a promising strategy for targeting circRNAs is emerging (Figure 3). Some ASOs that target circRNA are also complementary to pre-mRNAs, including introns. These ASOs can bind directly to pre-mRNA and cause its degradation, reducing the expression of mRNA and circRNA. Therefore, the target sequence of ASO should be carefully selected to avoid unwanted degradation of pre-mRNA. CRISPR/Cas13 systems have lower mismatch tolerance and inhibit circRNAs more specifically than RNAi. Recent research showed that CRISPR/Cas13 could mediate RNA-dependent RNA degradation in a programmable and site-specific manner.^78^ The technology could be helpful to directly target circRNAs at their unique splice junction, thereby circumventing the nonspecificity against the parental mRNA while providing better selectivity and safety. These studies have indicated that the CRISPR/Cas13 system may be one of the most promising therapeutic strategies for clinical cancer treatment, with circRNAs as the therapeutic target. Commonly used circRNA overexpression strategies include plasmid vector and viral vector delivery. The process of constructing a plasmid vector is relatively simple, but its application is limited due to its low transfection efficiency. Several viral vector tools can achieve efficient and stable transduction in various cells and animals. However, circRNA-expressing viral vectors can also cause unexpected side effects. Despite significant improvements in upstream and downstream processes, adeno-associated virus stocks still contain significant impurities that should be minimized during production. Otherwise, systemic administration of very high doses may result in immunotoxicity.^79^ Highly purified circRNA molecules synthesized *in vitro* may be used to overcome these shortcomings. However, artificially synthesized circRNAs are immunogenic, possibly due to the lack of the m6A modification of exogenous circRNAs.

## Bioinformatics Methodology in the Discovery of CircRNAs in Brain Tumor Patients

Moreover, advances in sequencing technologies and methods used in the discovery of circRNAs have dramatically improved the identification of circRNA abnormalities. Li et al. have proposed a serum exosome-based circRNA panel for diagnosis of high-grade astrocytoma (HGA) by circRNA profiling using RNA sequencing in HGA tumor and paired exosomes.^80^ Song et al. performed rRNA-depleted total RNA sequencing followed by bioinformatics analysis on 46 glioma and matched normal brain samples and found around 83% circRNA differentially expressed between normal and tumor brain samples.^81^ Gokool et al have created a comprehensive resource that catalogs the landscape of circRNA identification from large-scale brain sequencing studies.^82^ Similarly, MiOncoCirc houses compendium of circRNA identified from cancer sequencing studies of more than 2,000 patients and cell lines.^1^ Reports have suggested that some linear RNAs are resistant to RNase R treatment. This can give rise to erroneous readings in circRNA sequencing and interpretation of data. It was previously shown that due to structured 3ʹ tail of RNA or guanosine-rich regions in RNA can deter the processing of RNase R. Hence, novel methods have been developed for efficient enrichment of circRNA for sequencing.^83^ Additional poly(A) was done to significantly improve RNase R treatment and circRNA enrichment. Additionally, advanced platforms of sequencing, such as Oxford Nanopore, have now been employed for long-read-based circRNA transcript detection. circRNA Nicking and Long-Read Sequencing is a novel method for sequencing circRNA using nanopore sequencer. Briefly, rRNA-depleted total RNA is treated with RNase R to remove linear RNA. Resultant material is fragmented to linearize circRNA followed by 3ʹ phosphate group removal and 5ʹ phosphate group addition. Finally, linearized circRNA transcripts are poly(A) tailed for cDNA library preparation for nanopore sequencing. Similarly, variety of computational methods are available to detect and measure expression of circRNA from high-throughput sequencing datasets.^84^

## Conclusion and Future Prospects

The temporal, spatial, and tissue specificity of circRNA implies that circRNA is involved in the process of tumorigenesis and development. CircRNAs levels change along with angiogenesis, autophagy, apoptosis, tumorigenesis, and inflammation and are closely related to brain tumors. Increasing evidence shows that circRNAs can mediate the occurrence and progression of Glioma through miRNA sponging, transcriptional regulation, and protein interactions. These effects are obtained by operating critical molecular mechanisms or signaling pathways. Recent studies have found that m6A-modified circRNAs are usually derived from exons not methylated in mRNAs. The circRNAs from methylated mRNA exons are relatively unstable, and it is unclear whether m6A modification affects the stability of circRNAs. When circRNAs are degraded, the loop structure alters the properties of their corresponding linear RNAs. The functional implications of circRNAs have only been tentatively explored, probably due to the limitations of the research tools. Since circRNAs are highly stable and widely expressed in various tissues and body fluids, and they could be used as diagnostic and prognostic biomarkers. Further research is needed to explore the circRNAs associated with brain tumors. These circRNAs could be used in combination with traditional biological diagnostic indicators for clinical adjuvant screening of brain tumors at an early stage. Ultimately, this could improve the survival rate of brain tumor patients and achieve early detection and treatment. As more brain tumor-related and structurally diverse circRNAs are discovered, elucidating complex molecular regulatory mechanisms of brain tumors and applying circRNAs-based brain tumor diagnosis and treatment will have a broader prospect. For example, the new gold nanoparticle carrier could provide circRNA delivery, although its biological safety remains to be studied. Delivering ncRNAs to tumors remains challenging, but this is gradually being overcome through advanced technologies such as nanotechnology and rational biomaterial design. With further identification of the molecular mechanisms of circRNAs, especially the regulatory networks between circRNAs and other oncogenic and tumor suppressor genes, we postulate that circRNAs could soon serve as novel biomarkers and therapeutic targets for patients with brain tumors.

## List of unpublished papers cited

No unpublished data are referenced in this study.
